# IFI44L and C1QTNF5 as promising biomarkers of proliferative diabetic retinopathy

**DOI:** 10.1097/MD.0000000000031961

**Published:** 2022-11-25

**Authors:** Mingxin Shang, Yao Zhang, Tongtong Zhang

**Affiliations:** a He Eye Specialist Hospital, Shenyang, Liaoning Province, China.

**Keywords:** proliferative diabetic retinopathy, biomarker, IFI44L, C1QTNF5, LASSO, WGCNA

## Abstract

Proliferative diabetic retinopathy (PDR) is a world-wide leading cause of blindness among adults and may be associated with the influence of genetic factors. It is significant to search for genetic biomarkers of PDR. In our study, we collected genomic data about PDR from gene expression omnibus (GEO) database. Differentially expressed gene (DEG) analysis and weighted gene co-expression network analysis (WGCNA) were carried out. The gene module with the highest gene significance (GS) was defined as the key module. Hub genes were identified by Venn diagram. Then we verified the expression of hub genes in validation data sets and built a diagnostic model by least absolute shrinkage and selection operator (LASSO) regression. Enrichment analysis, including gene ontology (GO), Kyoto Encyclopedia of Genes and Genomes (KEGG), gene set enrichment analysis (GSEA) and construction of a protein–protein interaction (PPI) network were conducted. In GSE60436, we identified 466 DEGs. WGCNA established 14 gene modules, and the blue module (GS = 0.64), was the key module. Interferon (IFN)-induced protein 44-like (IFI44L) and complement C1q tumor necrosis factor-related protein 5 (C1QTNF5) were identified as hub genes. The expression of hub genes in GEO datasets was verified and a diagnostic model was constructed by LASSO as follows: index = IFI44L * 0.0432 + C1QTNF5 * 0.11246. IFI44L and C1QTNF5 might affect the disease progression of PDR by regulating metabolism-related and inflammatory pathways. IFI44L and C1QTNF5 may play important roles in the disease process of PDR, and a LASSO regression model suggested that the 2 genes could serve as promising biomarkers of PDR.

## 1. Introduction

Diabetes retinopathy (DR) is a major complication of diabetes and the leading cause of blindness in adults.^[1,2]^ As of 2019, there were about 463 million people with diabetes worldwide, and up to 35% of them have DR.^[3]^ Usually, DR is classified into 2 stages: non-proliferative diabetic retinopathy (NPDR) and proliferative diabetic retinopathy (PDR).^[4]^ Some known risk factors for DR include the duration of diabetes, hyperglycemia, hypertension, and hyperlipidemia. By controlling these risk factors, many patients with DR could be effectively prevented from developing into the sight-threatening type of PDR or neovascular glaucoma (NVG).^[5]^ However, there is still a high proportion of patients with DR whose disease progression is difficult to control. The pathogenesis of PDR is complex and involves vascular, inflammatory, and neuronal mechanisms.^[6]^ These may be closely associated with the influence of genetic factors.^[7]^

With the rapid development of genomics, many genes have been found to be related to PDR, such as vascular endothelial growth factor (VEGF),^[8]^ hypoxia-inducible factor 1-α (HIF1A),^[9]^ erythropoietin (EPO),^[10]^ receptor for advanced glycation end product (RAGE),^[11]^ among others. Neovascularization is an important feature of PDR, thus anti-VEGF agents have become a promising therapy for PDR.^[12]^ So far, anti-VEGF therapy has shown poor efficacy for diabetic macular edema (DME), another major factor affecting the prognosis of PDR,^[13]^ and this phenomenon is likely to be related to individual genetic differences. Therefore, it is of great significance to search for genetic biomarkers of PDR.

The development of bioinformatics plays a powerful role in promoting genomics research. Gene expression omnibus (GEO, https://www.ncbi.nlm.nih.gov/geo/) is a public database that contains microarray and high-throughput genomic datasets. It also includes genomic data from human patients with PDR.^[14–17]^ Weighted gene co-expression network analysis (WGCNA) is a well-known systems biology method to identify gene association patterns.^[18]^ Co-expressed genes are clustered into various modules named after colors. These modules are correlated with phenotypic traits, and key genes within the networks should be found.^[19]^ Least absolute shrinkage and selection operator (LASSO) is an effective approach for the regression of high-dimensional parameters and has been broadly applied for diagnostic and prognostic analysis.^[20,21]^

The objective of our research was to identify genomic biomarkers for PDR. Firstly, we searched the GEO database for genomic data of human patients with PDR. Comprehensive bioinformatics approaches, including WGCNA, were utilized to construct a gene co-expression network (GSE60436), find the key gene module and the hub genes, verify the expression of hub genes in validation datasets (GSE94019, GSE160306 and GSE178721) and analyze the enriched pathways of genes in the key module. Finally, the LASSO regression model was adopted to investigate the diagnostic value of hub genes for PDR.

## 2. Materials and methods

### 2.1. Data collection and pre-processing

By searching the GEO database using the key words “proliferative diabetic retinopathy,” we have found 4 datasets, including GSE60436, GSE94019, GSE160306 and GSE178721. GSE60436 included 6 tissues of PDR and 3 normal tissue samples, which were based on GPL6884 (Illumina HumanWG-6 v3.0 expression beadchip).^[14]^ GSE94019 contained 9 PDR tissues and 4 normal tissues based on GPL11154 (Illumina HiSeq 2000).^[15]^ GSE160306 included 5 PDR with DME tissues, 15 NPDR with DME tissues, 19 NPDR tissues, 20 diabetic tissues, and 20 normal tissues based on GPL20301 (Illumina HiSeq 4000).^[16]^ GSE178721 contained 1 PDR serum sample and 1 macular hole (MH) sample based on GPL21290 (Illumina HiSeq 3000, Illumina, San Diego, CA). ^[17]^

Expression profiles of 4 datasets were downloaded. Raw expression data of GSE60436 was obtained and further normalized by the “lumi” package in the R language.^[22]^ Gene reads or gene counts data were acquired and transferred into counts per million (CPM) matrices using the R package “edgeR.”^[23]^

### 2.2. Screening for differentially expressed genes (DEGs)

We utilized the R package “limma” to identify the DEGs,^[24]^ by setting the statistical threshold to *P* < .05 and the absolute value of fold change (FC) > 2. Volcano plots and heatmaps were drawn by the “ggplot 2” and “ggpubr” packages in R.

### 2.3. Weighted gene co-expression network construction

The WGCNA package in R was used to build a gene co-expression network for GSE64036.^[18]^ We picked out 5000 genes with the largest median values at the beginning. And then we selected a soft threshold using the “pickSoftThreshold” function of the package. Afterwards, genes were classified into multiple gene modules. The gene module with the highest gene significance (GS) value was defined as the key module. Genes in the key module with module membership (MM) > 0.9 and GS > 0.3 were considered interesting genes.

### 2.4. Identification and verification of hub genes

Hub genes were identified by the overlapping of DEGs in GSE60436 and interesting genes in the key module. And we further verified the expression of hub genes in GSE94019, GSE160306 and GSE178721. We generated Venn diagrams using the Venny tool (https://bioinfogp.cnb.csic.es/tools/venny/index.html) and generated violin plots and box plots using the R packages “ggstatsplot” and “ggpubr.”

### 2.5. Functional enrichment analysis of key module

Gene ontology (GO) enrichment analysis and Kyoto Encyclopedia of Genes and Genomes (KEGG) pathway analysis of genes in the key module were performed using the R package “ClusterProfiler.”^[25]^ Gene set enrichment analysis (GSEA) and gene set variation analysis (GSVA) are popular tools for analyzing pathways enriched in gene expression profiles. We ran GSVA analysis of GSE60436 by the “GSVA” package in R.^[26]^ Additionally, samples from GSE94019 were divided into high-expression groups and low-expression groups according to the expression levels of single hub genes, and GSEA analysis was performed by the “ClusterProfiler” package.

### 2.6. Construction of a protein–protein interaction (PPI) network

Genes in the key module were imported into the STRING database (https://string-db.org/).^[27]^ A PPI network was constructed in the Cytoscape software.^[28]^ And we calculated and visualized the Maximum Neighborhood Component (MCC) value of each node in the PPI network by the “Cytohubba” plugin.^[29]^

### 2.7. Construction of LASSO model and receiver operating characteristic (ROC) curve analysis

Firstly, GSE94019 and GSE160306 were combined and then randomly assigned to the training set (70%) and the test set (30%). Then we constructed the LASSO model by using the “glmnet” package in R.^[30]^ Using the regression coefficients from LASSO, a model index to weight the expression of hub genes was established with the formula: index = Gene1 * Coefficient1 + Gene2 * Coefficient2. R package “pROC” was utilized to generate ROC curve diagrams in the training set and the test set.^[31]^

## 3. Results

### 3.1. Identification of DEGs in PDR

**Figure 1.**
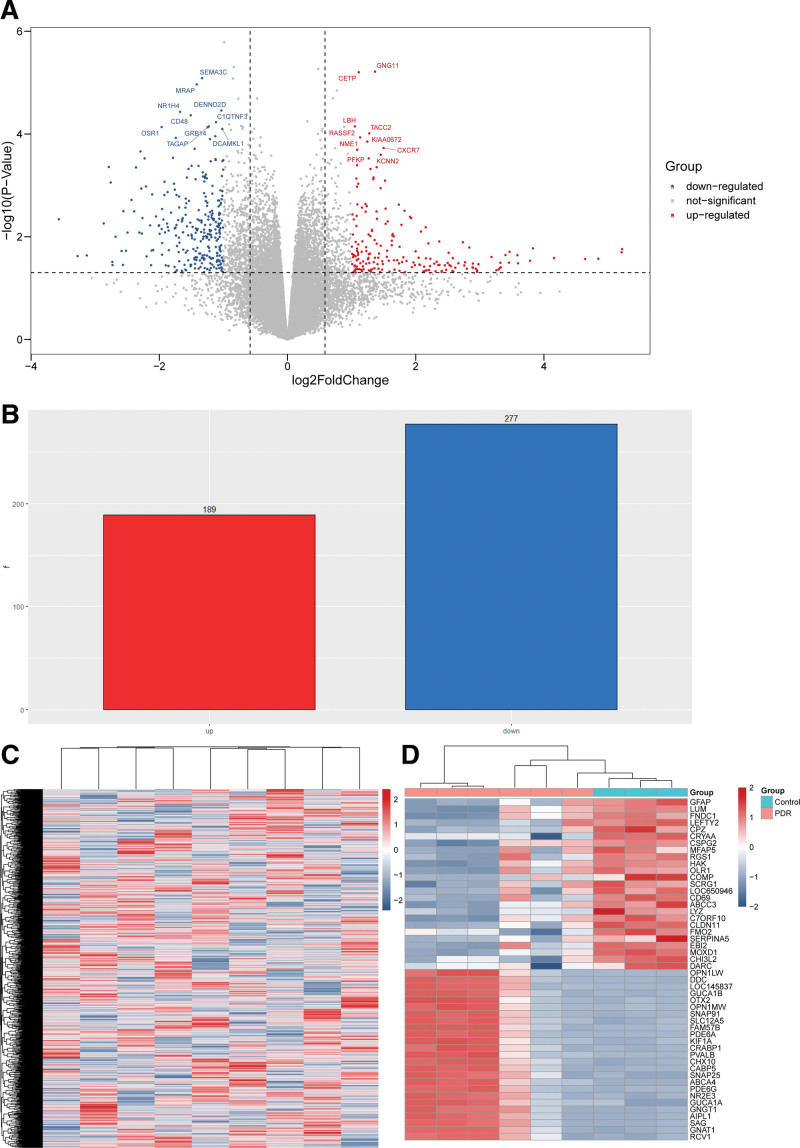
Differentially expressed genes (DEGs) present in the proliferative diabetic retinopathy (PDR) and control groups in GSE60436. (A) Volcano plot showed the distribution of the DEGs between 2 groups. The red and blue dots correspond to the upregulated and downregulated genes, respectively. (B) Bar plot showed the number of upregulated DEGs (red) and downregulated DEGs (blue). (C) Heatmap of the overall gene expression in GSE60436. Red indicates the upregulated genes and blue indicates the downregulated genes. (D) Heatmap of the top 25 upregulated DEGs (red) and the top 25 downregulated DEGs (blue) ranked by fold change.

In GSE60436, there were 466 DEGs in the PDR tissue samples compared to control samples, including 189 upregulated genes (RCV1, GNAT1, SAG, AIPL1, GNGT1, among others) and 277 downregulated genes (GFAP, LUM, FNDC1, LEFTY2, CPZ, among others). The results of the DEG analysis were represented as volcano plots and heatmaps (Fig. 1).

### 3.2. Identification of the key gene module by WGCNA

**Figure 2.**
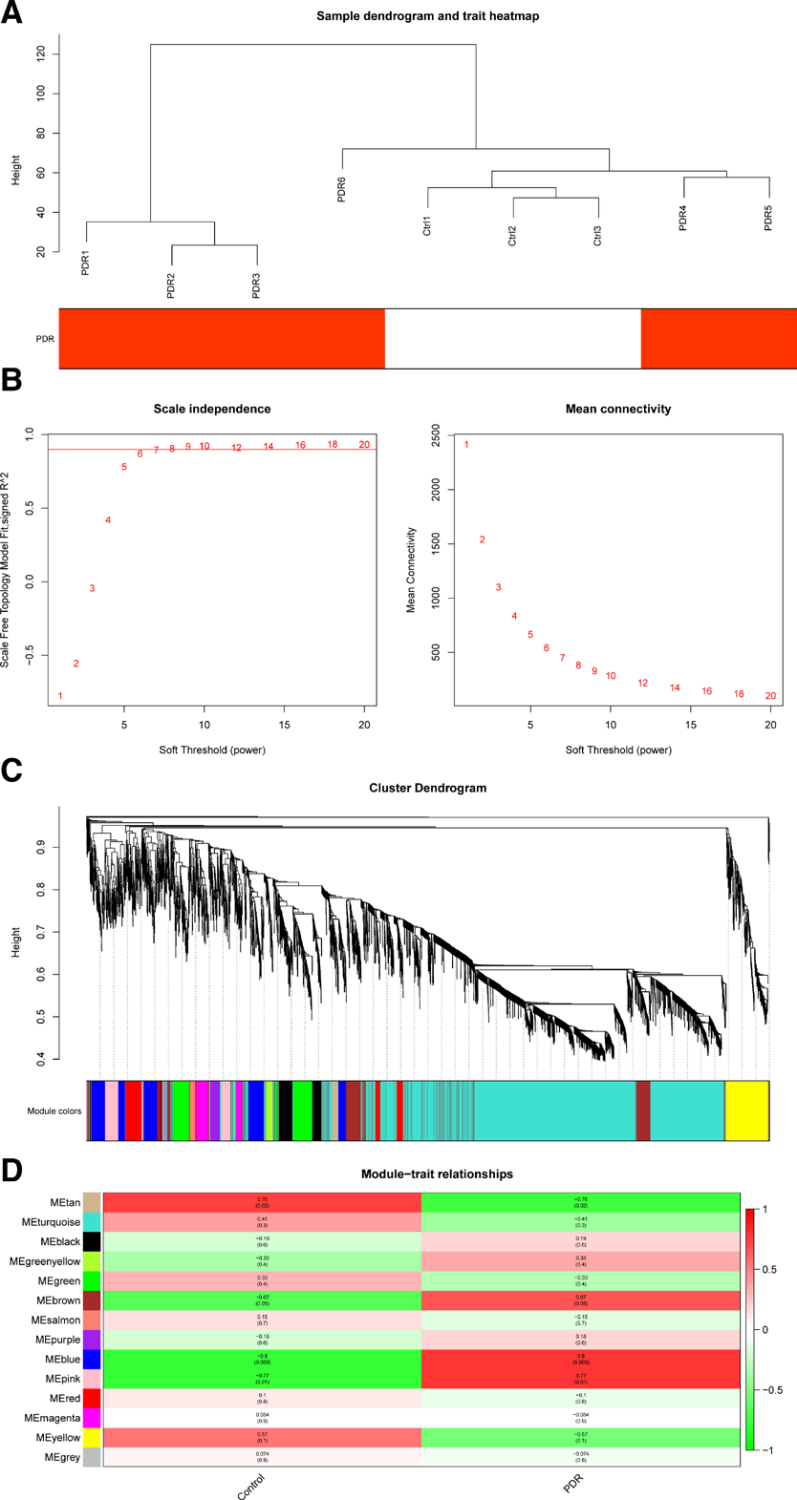
Gene co-expression network construction by weighted gene co-expression network analysis (WGCNA). (A) Sample data clustering showed no obvious outliers found. Red color represents the proliferative diabetic retinopathy (PDR) group. Ctrl, control group. (B) Determination of soft threshold. Left: scale independence; right: mean connectivity. (C) Cluster dendrogram. Each color represents 1 specific gene module; the above branches represent genes. (D) Module-trait relationships among the 14 gene modules. The GS value of the blue module was the highest. GS = gene significance.

**Figure 3.**
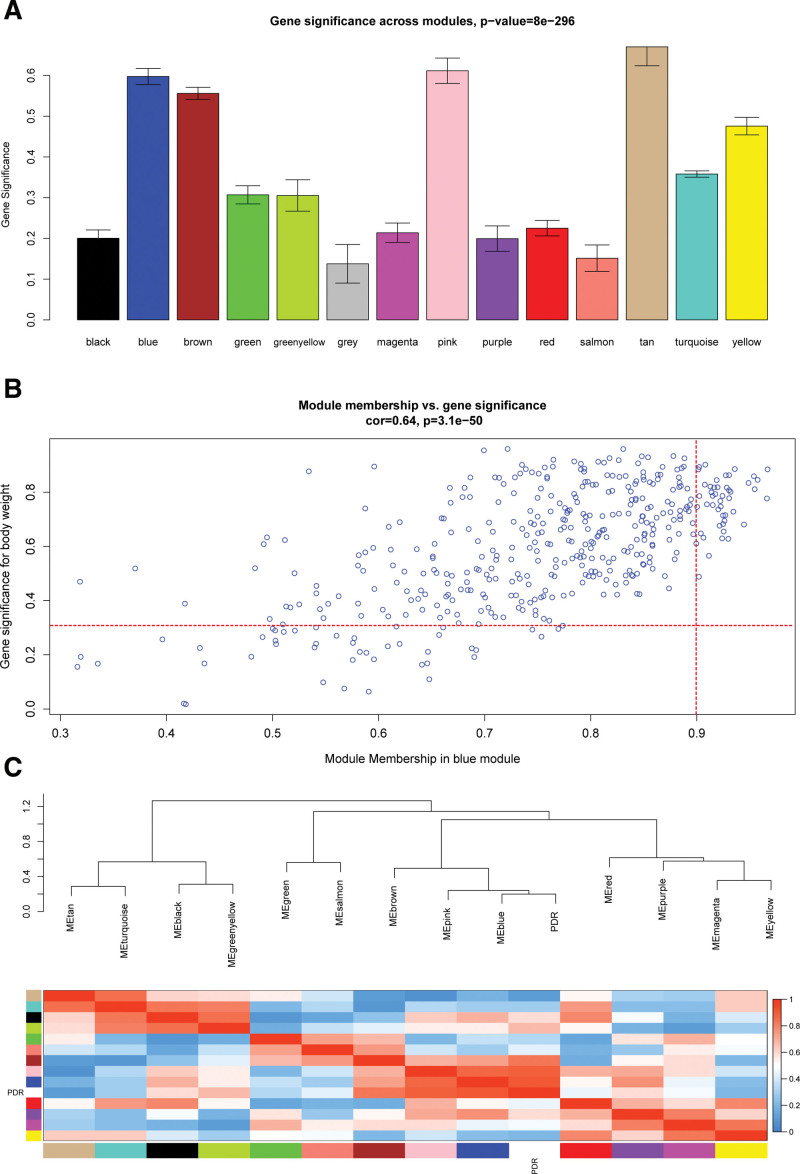
Screening of interesting genes in the key module. (A) Gene significance (GS) across modules. (B) Module membership (MM) vs. GS in the blue module. The red dotted lines represent the thresholds of MM > 0.9 and GS > 0.3 set for interesting genes. (C) Eigengene dendrogram and adjacency heatmap displayed the correlation between proliferative diabetic retinopathy (PDR) and the modules.

Firstly, we screened the top 5000 genes with the highest median to run WGCNA. Then, sample data was clustered, and no outliers were found in our study (Fig. 2A). The soft threshold was set to 6 according to the “pickSoftThreshold” function (Fig. 2B). Then a total of 14 gene modules were generated based on WGCNA (Fig. 2C, and see Table S1, Supplemental Digital Content, http://links.lww.com/MD/H995, which illustrates the statistic of gene modules constructed by WGCNA). The modules were named after different colors. The largest turquoise module was composed of 2568 genes and the smallest gray module was composed of 19 genes. The blue module scored the highest among the gene modules (GS = 0.64, (Figs. 2D, 3A and 3B), so it was identified as the key module. In the blue module, 46 interesting genes whose module membership (MM) > 0.9 and GS > 0.3 were picked out (Fig. 3B). The eigengene dendrogram and adjacency heatmap displayed the correlation between PDR and the modules (Fig. 3C).

### 3.3. Identification and verification of hub genes

**Figure 4.**
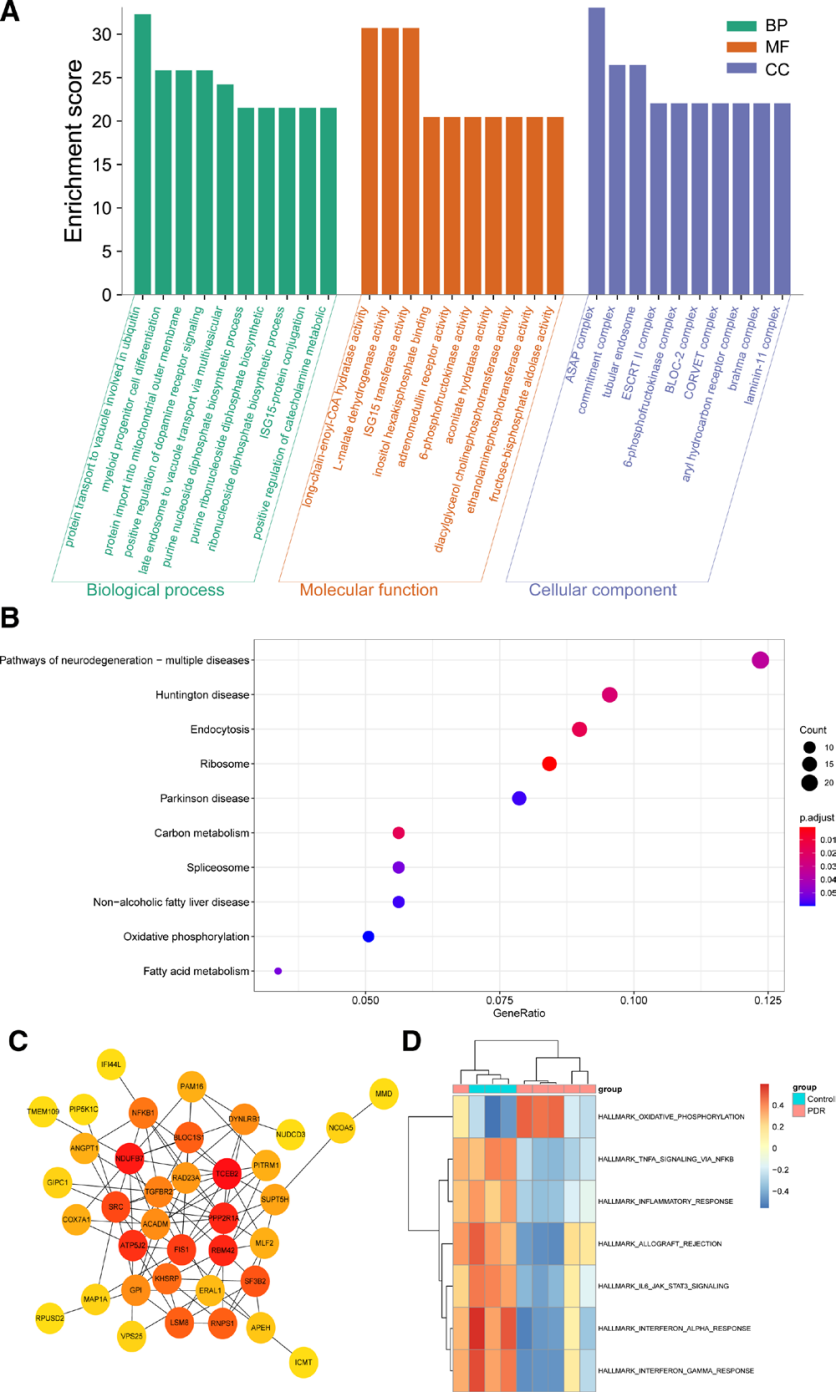
Identification and expressions of hub genes in GSE60436, GSE94019, GSE160306 and GSE178721. (A) Venn diagram showed IFI44L and C1QTNF5 in the intersection of 46 interesting genes in the blue module and 466 differentially expressed genes (DEGs) in GSE60436. (B) Violin plots of the expressions of IFI44L and C1QTNF5 in GSE60436. X-axis represented the control group and the proliferative diabetic retinopathy (PDR) group. Y-axis represented the expression of genes. (C) Violin plots of the expressions of IFI44L and C1QTNF5 in GSE94019. (D) Violin plots of the expressions of IFI44L in GSE160306. X-axis represented the control group, diabetic group, non-proliferative diabetic retinopathy (NPDR) group, NPDR with diabetic macular edema (DME) group and PDR with DME group. (E) Violin plots of the expressions of C1QTNF5 in GSE160306. (F) Box plots of the expressions of IFI44L and C1QTNF5 in GSE178721. The green box represented PDR group and the red box represented the control group. **P < .01; *P < .05. C1QTNF5 = complement C1q tumor necrosis factor-related protein 5, IFI44L = interferon (IFN)-induced protein 44-like.

By taking the intersection of DEGs in GSE60436 and interesting genes in the blue module, interferon (IFN)-induced protein 44-like (IFI44L) and complement C1q tumor necrosis factor-related protein 5 (C1QTNF5) were identified as hub genes (Fig. 4A). We verified the expression of IFI44L and C1QTNF5 in GSE94019, GSE160306, and GSE178721 (Fig. 4B–F). IFI44L was found highly expressed in GSE94019, GSE160306, and GSE178721 but not in GSE60436. C1QTNF5 was highly expressed in all 4 datasets but was not significantly different in GSE160306 and GSE178721. What’s more, we have discovered that high IFI44L expression seemed to have strong associations with the disease status of diabetic retinopathy according to its expression in GSE160306. More importantly, similar trends in IFI44L expression were shown in the tissue samples (GSE94019 and GSE160306) and serum samples (GSE178721).

### 3.4. Enriched biological processes and pathways in the key module and PPI network construction

**Figure 5.**
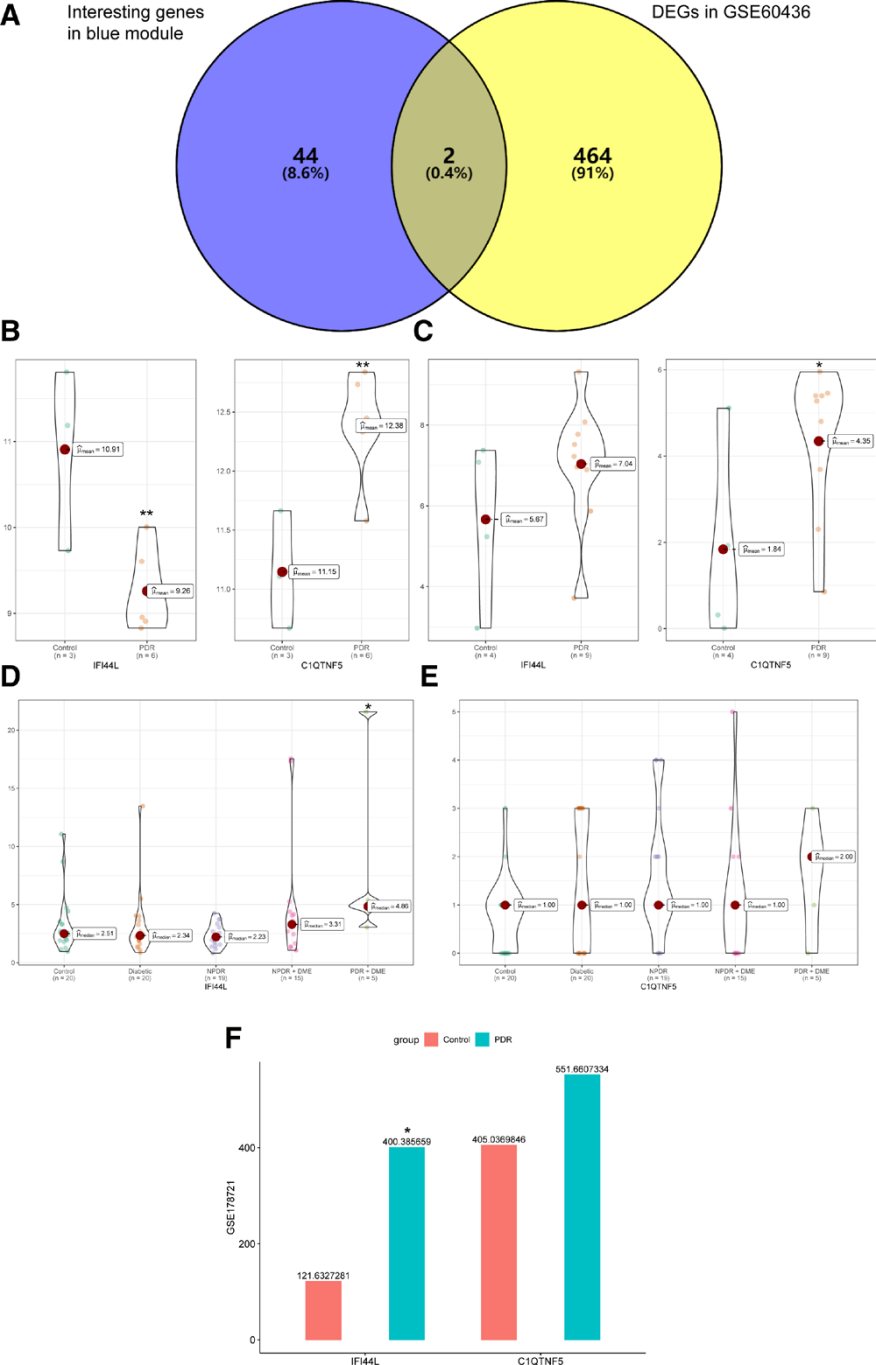
Functional enrichment analysis of genes in the blue module. (A) Gene ontology (GO) terms enrichment analysis of the blue module. Top 10 terms in GO biological process (BP), molecular function (MF) and cellular component (CC) enrichment analysis, sorted by the enrichment score. (B) Significantly enriched KEGG pathways in the blue module, gene count, and –log10 (*P*-value). (C) Protein-protein interaction network of interesting genes in the blue module, the dots represent proteins, the change of their colors from red to yellow reflect their Maximum Neighborhood Component (MCC) value calculated by “Cytohubba” plugin from high to low, and the lines represent protein interactions. (D) Heatmap of gene set variation analysis (GSVA) in GSE60436, red indicates the upregulated gene sets and blue indicates the downregulated gene sets. KEGG = Kyoto encyclopedia of genes and genomes.

**Figure 6.**
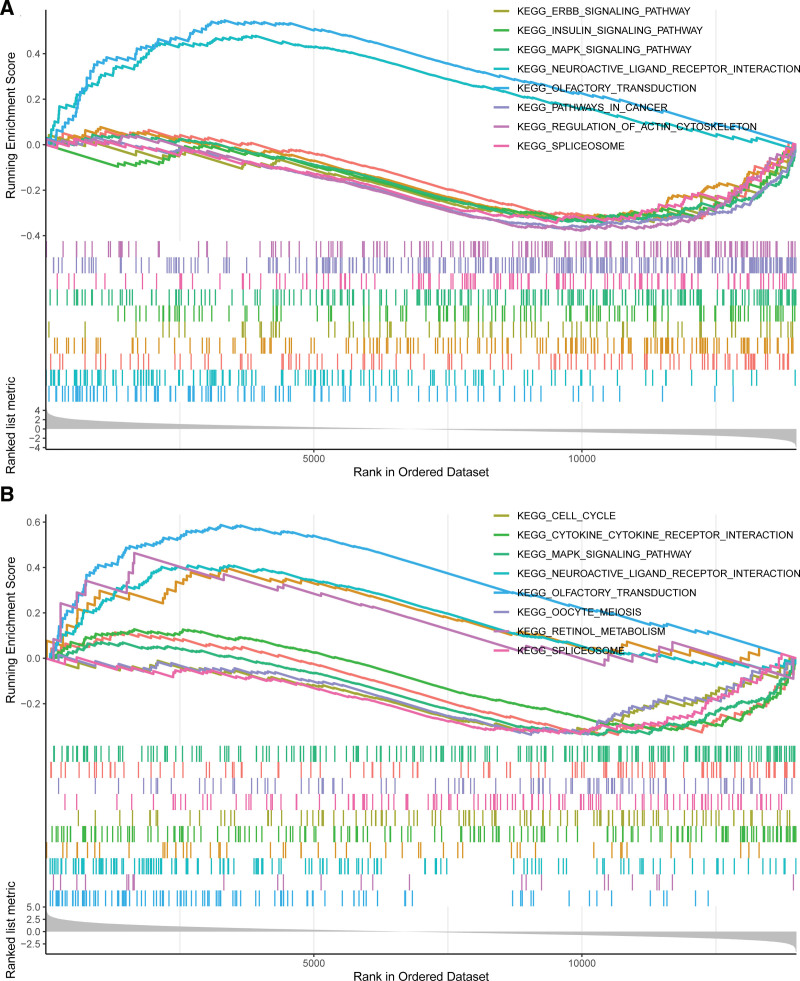
Gene set enrichment analysis (GSEA) of hub genes in GSE94019. (A) Top 8 gene sets (according to GSEA enrichment score) enriched in the IFI44L high-expressed group. (B) Top 8 gene sets enriched in the C1QTNF5 high-expressed group. C1QTNF5 = complement C1q tumor necrosis factor-related protein 5, IFI44L = interferon (IFN)-induced protein 44-like.

Genes in the blue module were mainly enriched in protein transport to the vacuole involved in ubiquitin-dependent protein catabolic process via the multivesicular body sorting pathway (GO:0043328) in GO biological process, long chain enoyl-CoA hydratase activity (GO:0016508) in GO molecular function, and 6-phosphofructokinase complex (GO:0005945) in GO cellular component (Fig. 5A), and see Table S2, Supplemental Digital Content, http://links.lww.com/MD/H996, which illustrates GO terms enrichment analysis of the blue module). Kyoto Encyclopedia of Genes and Genomes (KEGG) pathway analysis showed that pathways of neurodegeneration-multiple diseases (hsa05022) and endocytosis (hsa04144) were regulated by the blue module genes (Fig. 5B). Moreover, a PPI network was constructed for interesting genes in the blue module, which consisted of 38 gene nodes (Fig. 5C). GSVA of GSE60436 suggested the importance of the TNF-α signaling pathway and interferon α response for PDR (Fig. 5D). According to GSEA results of GSE94019, IFI44L might act on the insulin signaling pathway and mitogen‐activated protein kinase (MAPK) signaling pathway (Fig. 6A) and C1QTNF5 might affect the retinol metabolism pathway and MAPK signaling pathway (Fig. 6B).

### 3.5. LASSO model of IFI44L and C1QTNF5

**Figure 7.**
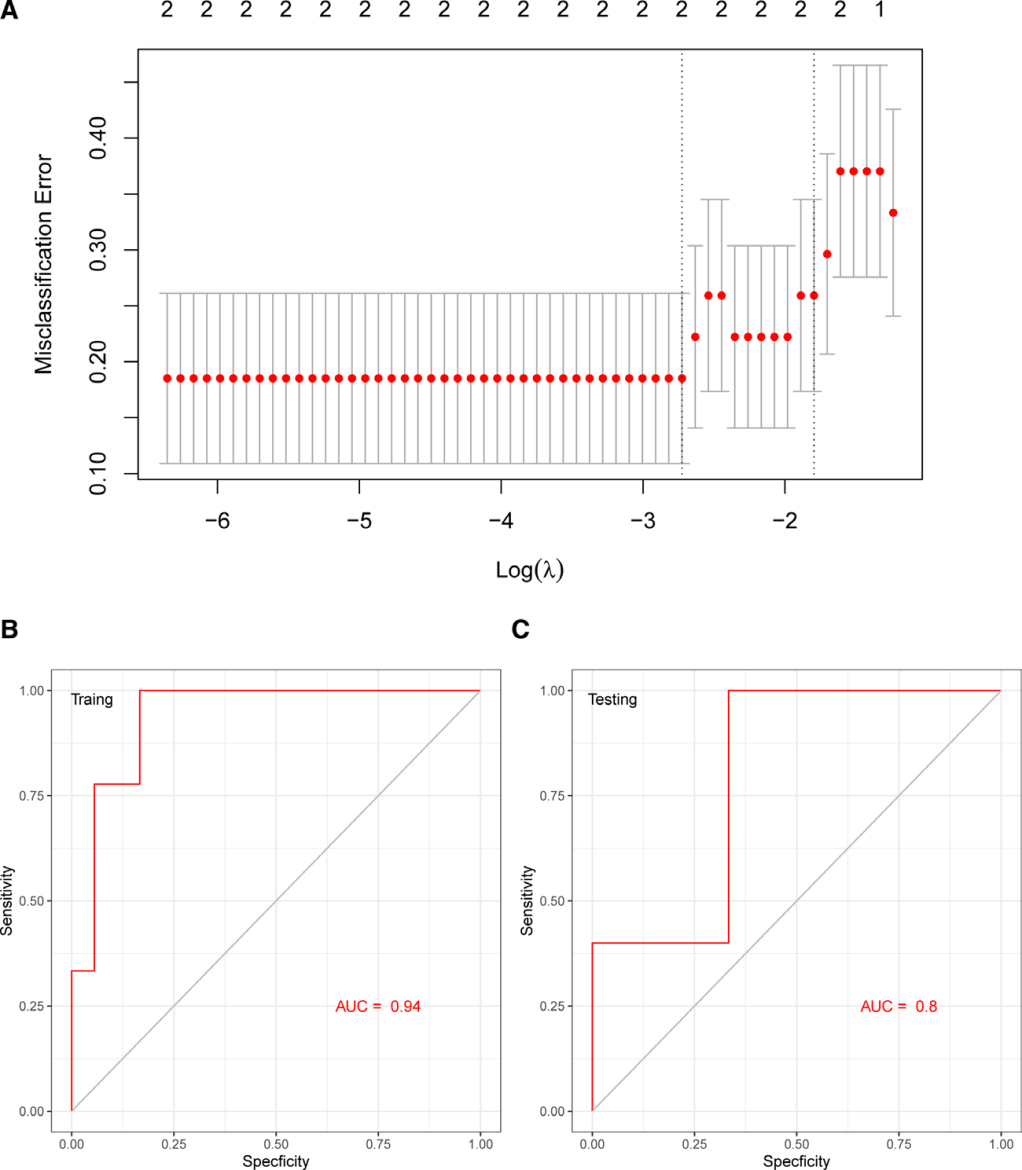
Least absolute shrinkage and selection operator (LASSO) model for predicting PDR and receiver operating characteristic (ROC) curve analysis. (A) LASSO model. (B) ROC curves analysis of training set (GSE94019 and GSE160306 were combined and then 70% of the combined dataset were randomly assigned to the training set). (C) ROC curves analysis of test set (the rest 30% of the combined dataset were assigned to the test set). PDR = proliferative diabetic retinopathy.

We utilized the LASSO model to identify IFI44L and C1QTNF5 with non-zero regression coefficients. The minimum value of lambda was 0.001245652. Two gene-based model indexes were created as follows: index = IFI44L * 0.0432 + C1QTNF5 * 0.11246 (Fig. 7A). ROC curve analysis indicated that the area under the curve (AUC) of the two-gene-based model was 0.94 in the training set (Fig. 7B) and 0.80 in the test set (Fig. 7C). This suggested that 2 hub genes could serve as potential diagnostic biomarkers of PDR.

## 4. Discussion

Various proteins, cytokines, deoxyribonucleic acid (DNA) methylation, messenger ribonucleic acid (mRNA), and non-coding RNAs are considered as biomarkers for PDR, for instance, CCDC144NL and ZNF80,^[32]^ MMP-14,^[33]^ extracellular matrix metalloproteinase inducer (EMMPRIN),^[34]^ miR-296,^[35]^ miR-20b, miR-17-3p, HOTAIR, and MALAT1,^[36]^ hsa_circ_0001953.^[37]^ The relationships between IFI44L and C1QTNF5 with PDR have not been reported yet.

In our study, GSE60436 was divided into 14 modules by WGCNA, among which the enriched GO functions of the key module involved a variety of glucose and lipid metabolic pathways similar to those reported.^[38,39]^

We then identified IFI44L and C1QTNF5 as hub genes and verified their expression in validation data sets. Finally, a diagnostic model was established by LASSO regression, and the diagnostic significance of the 2 genes for PDR was further analyzed.

The IFI44L gene, is an important paralog of the IFI44 gene. It has been reported that IFI44L is closely associated with tuberculosis,^[40]^ Zika virus infection,^[41]^ hepatitis C^[42]^ and hepatocellular carcinoma.^[43]^ In a study of human retinal pigmented epithelial cells (RPE) in an age-related macular degeneration (AMD) disease model, IFI44L was significantly differentially expressed and may play a role in regulating inflammatory pathways.^[44]^

Late-onset retinal degeneration has been linked to mutations in the C1QTNF5 gene.^[45,46]^ It belongs to the C1q/TNF-related protein (CTRP) family, a family of genes that link immunity to metabolism by exerting anti-inflammatory and insulin-sensitizing effects.^[47]^ Surprisingly, C1q/TNF-related protein 3 (CTRP3) in the same family might serve as a novel biomarker for DR severity.^[48]^

The pathogenesis of PDR is related to inflammatory mechanisms and retinol and insulin metabolism.^[6,49,50]^ GSEA suggested that IFI44L could affect the insulin signaling pathway and the MAPK signaling pathway, and that C1QTNF5 could affect the retinol metabolism pathway and the MAPK signaling pathway. The MAPK signaling pathway is among the well-known cellular inflammatory pathways.^[51]^ Therefore, we might hypothesize that IFI44L and C1QTNF5 might affect the disease progression of PDR by regulating metabolism-related pathways and inflammatory pathways.

However, there were several shortcomings in our study. Our study was based on genomic data analysis, but the sample size of public datasets is limited. And 2 hub genes were not statistically significant in all the validation datasets. Moreover, our study was merely a theoretical analysis without experimental verification. In the follow-up study, we will expand the sample size of clinical patients to verify the difference in hub genes in peripheral blood samples and explore the possible mechanism of hub genes in PDR through experiments.

## 5. Conclusion

IFI44L and C1QTNF5 may play important roles in the disease process of PDR, and a LASSO regression model suggested that the 2 genes could serve as promising biomarkers of PDR.

## Acknowledgements

We acknowledge the GEO database for providing their platforms and contributors for uploading their meaningful datasets.

## Author contributions

**Conceptualization**: Tongtong Zhang.

**Data curation**: Mingxin Shang, Yao Zhang.

**Formal analysis**: Mingxin Shang.

**Funding acquisition**: Tongtong Zhang.

**Investigation**: Yao Zhang.

**Methodology**: Mingxin Shang.

**Software**: Mingxin Shang.

**Supervision**: Tongtong Zhang.

**Writing – original draft**: Mingxin Shang.

**Writing – review & editing**: Tongtong Zhang.

All authors reviewed the manuscript.

## Supplementary Material

## References

[1] Joussen AM Poulaki V Le ML. A central role for inflammation in the pathogenesis of diabetic retinopathy. FASEB J. 2004;18:1450–2. 15231732 10.1096/fj.03-1476fje

[2] Yau JW Rogers SL Kawasaki R. Global prevalence and major risk factors of diabetic retinopathy. Diabetes Care. 2012;35:556–64. 22301125 10.2337/dc11-1909 PMC3322721

[3] Saeedi P Petersohn I Salpea P. Global and regional diabetes prevalence estimates for 2019 and projections for 2030 and 2045: results from the International Diabetes Federation Diabetes Atlas, 9(th) edition. Diabetes Res Clin Pract. 2019;157:107843. 31518657 10.1016/j.diabres.2019.107843

[4] Gu C Lhamo T Zou C. Comprehensive analysis of angiogenesis-related genes and pathways in early diabetic retinopathy. BMC Med Genomics. 2020;13:142. 32993645 10.1186/s12920-020-00799-6 PMC7526206

[5] Wong TY Cheung CMG Larsen M. Diabetic retinopathy. Nat Rev Dis Primers. 2016;2:16012. 27159554 10.1038/nrdp.2016.12

[6] Rezzola S Loda A Corsini M. Angiogenesis-inflammation cross talk in diabetic retinopathy: novel insights from the chick embryo chorioallantoic membrane/human vitreous platform. Front Immunol. 2020;11:581288. 33117388 10.3389/fimmu.2020.581288 PMC7552803

[7] Lachin JM Genuth S Nathan DM. Effect of glycemic exposure on the risk of microvascular complications in the diabetes control and complications trial – revisited. Diabetes. 2008;57:995–1001. 18223010 10.2337/db07-1618

[8] Abhary S Burdon KP Gupta A. Common sequence variation in the VEGFA gene predicts risk of diabetic retinopathy. Invest Ophthalmol Vis Sci. 2009;50:5552–8. 19553626 10.1167/iovs.09-3694

[9] Loukovaara S Koivunen P Inglés M. Elevated protein carbonyl and HIF-1α levels in eyes with proliferative diabetic retinopathy. Acta Ophthalmol. 2014;92:323–7. 23718695 10.1111/aos.12186

[10] Song Q Zhang Y Wu Y. Association of erythropoietin gene polymorphisms with retinopathy in a Chinese cohort with type 2 diabetes mellitus. Clin Exp Ophthalmol. 2015;43:544–9. 25675872 10.1111/ceo.12505

[11] Kerkeni M Saïdi A Bouzidi H. Elevated serum levels of AGEs, sRAGE, and pentosidine in Tunisian patients with severity of diabetic retinopathy. Microvasc Res. 2012;84:378–83. 22835520 10.1016/j.mvr.2012.07.006

[12] Zhang Y Gao Z Zhang X. Effect of intravitreal conbercept injection on VEGF-A and -B levels in the aqueous and vitreous humor of patients with proliferative diabetic retinopathy. Exp Ther Med. 2021;21:332. 33732305 10.3892/etm.2021.9763 PMC7903486

[13] Jampol LM Bressler NM Glassman AR. Revolution to a new standard treatment of diabetic macular edema. JAMA. 2014;311:2269–70. 24915254 10.1001/jama.2014.2536 PMC4222042

[14] Ishikawa K Yoshida S Kobayashi Y. Microarray analysis of gene expression in fibrovascular membranes excised from patients with proliferative diabetic retinopathy. Invest Ophthalmol Vis Sci. 2015;56:932–46. 25604687 10.1167/iovs.14-15589

[15] Lam JD Oh DJ Wong LL. Identification of RUNX1 as a mediator of aberrant retinal angiogenesis. Diabetes. 2017;66:1950–6. 28400392 10.2337/db16-1035 PMC5482092

[16] Becker K Klein H Simon E. In-depth transcriptomic analysis of human retina reveals molecular mechanisms underlying diabetic retinopathy. Sci Rep. 2021;11:10494. 34006945 10.1038/s41598-021-88698-3 PMC8131353

[17] Wang YY Zhang H-Y Jiang W-J. Genetic polymorphisms in pri-let-7a-2 are associated with ischemic stroke risk in a Chinese Han population from Liaoning, China: a case-control study. Neural Regen Res. 2021;16:1302–7. 33318409 10.4103/1673-5374.301019 PMC8284288

[18] Langfelder P Horvath S. WGCNA: an R package for weighted correlation network analysis. BMC Bioinf. 2008;9:559. 10.1186/1471-2105-9-559 PMC2631488 19114008

[19] Langfelder P Zhang B Horvath S. Defining clusters from a hierarchical cluster tree: the dynamic tree cut package for R. Bioinformatics. 2008;24:719–20. 18024473 10.1093/bioinformatics/btm563

[20] Tibshirani R. The lasso method for variable selection in the Cox model. Stat Med. 1997;16:385–95. 9044528 10.1002/(sici)1097-0258(19970228)16:4<385::aid-sim380>3.0.co;2-3

[21] Sauerbrei W Royston P Binder H. Selection of important variables and determination of functional form for continuous predictors in multivariable model building. Stat Med. 2007;26:5512–28. 18058845 10.1002/sim.3148

[22] Du P Kibbe WA Lin SM. lumi: a pipeline for processing Illumina microarray. Bioinformatics. 2008;24:1547–8. 18467348 10.1093/bioinformatics/btn224

[23] Robinson MD McCarthy DJ Smyth GK. edgeR: a Bioconductor package for differential expression analysis of digital gene expression data. Bioinformatics. 2010;26:139–40. 19910308 10.1093/bioinformatics/btp616 PMC2796818

[24] Ritchie ME Phipson B Wu D. limma powers differential expression analyses for RNA-sequencing and microarray studies. Nucleic Acids Res. 2015;43:e47. 25605792 10.1093/nar/gkv007 PMC4402510

[25] Yu G Wang L-G Han Y. clusterProfiler: an R package for comparing biological themes among gene clusters. OMICS J Integr Biol. 2012;16:284–7. 10.1089/omi.2011.0118 PMC3339379 22455463

[26] Hänzelmann S Castelo R Guinney J. GSVA: gene set variation analysis for microarray and RNA-seq data. BMC Bioinf. 2013;14:7. 10.1186/1471-2105-14-7 PMC3618321 23323831

[27] Szklarczyk D Gable AL Lyon D. STRING v11: protein-protein association networks with increased coverage, supporting functional discovery in genome-wide experimental datasets. Nucleic Acids Res. 2019;47:D607–13. 30476243 10.1093/nar/gky1131 PMC6323986

[28] Shannon P Markiel A Ozier O. Cytoscape: a software environment for integrated models of biomolecular interaction networks. Genome Res. 2003;13:2498–504. 14597658 10.1101/gr.1239303 PMC403769

[29] Chin CH Chen S-H Wu H-H. cytoHubba: identifying hub objects and sub-networks from complex interactome. BMC Syst Biol. 2014;8(Suppl 4):S11. 25521941 10.1186/1752-0509-8-S4-S11 PMC4290687

[30] Engebretsen S Bohlin J. Statistical predictions with glmnet. Clin Epigenetics. 2019;11:123. 31443682 10.1186/s13148-019-0730-1 PMC6708235

[31] Robin X Turck N Hainard A. pROC: an open-source package for R and S+ to analyze and compare ROC curves. BMC Bioinf. 2011;12:77. 10.1186/1471-2105-12-77 PMC3068975 21414208

[32] Pan J Liu S Farkas M. Serum molecular signature for proliferative diabetic retinopathy in Saudi patients with type 2 diabetes. Mol Vis. 2016;22:636–45. 27307695 PMC4902182

[33] Abu El-Asrar AM Mohammad G Allegaert E. Matrix metalloproteinase-14 is a biomarker of angiogenic activity in proliferative diabetic retinopathy. Mol Vis. 2018;24:394–406. 29853773 PMC5957543

[34] Abu El-Asrar AM Ahmad A Alam K. Extracellular matrix metalloproteinase inducer (EMMPRIN) is a potential biomarker of angiogenesis in proliferative diabetic retinopathy. Acta Ophthalmol. 2017;95:697–704. 27860331 10.1111/aos.13284

[35] Smit-McBride Z Nguyen AT Yu AK. Unique molecular signatures of microRNAs in ocular fluids and plasma in diabetic retinopathy. PLoS One. 2020;15:e0235541. 32692745 10.1371/journal.pone.0235541 PMC7373301

[36] Shaker OG Abdelaleem OO Mahmoud RH. Diagnostic and prognostic role of serum miR-20b, miR-17-3p, HOTAIR, and MALAT1 in diabetic retinopathy. IUBMB Life. 2019;71:310–20. 30468285 10.1002/iub.1970

[37] Wu Z Liu B Ma Y. Discovery and validation of hsa_circ_0001953 as a potential biomarker for proliferative diabetic retinopathy in human blood. Acta Ophthalmol. 2021;99:306–13. 32914551 10.1111/aos.14585

[38] Tyni T Palotie A Viinikka L. Long-chain 3-hydroxyacyl-coenzyme A dehydrogenase deficiency with the G1528C mutation: clinical presentation of thirteen patients. J Pediatr. 1997;130:67–76. 9003853 10.1016/s0022-3476(97)70312-3

[39] Liu Z Xu J Ma Q. Glycolysis links reciprocal activation of myeloid cells and endothelial cells in the retinal angiogenic niche. Sci Transl Med. 2020;12:eaay1371. 32759274 10.1126/scitranslmed.aay1371 PMC7751280

[40] Liu S Ren W Yu J. Identification of hub genes associated with diabetes mellitus and tuberculosis using bioinformatic analysis. Int J Gen Med. 2021;14:4061–72. 34354368 10.2147/IJGM.S318071 PMC8331204

[41] Robinson CL Chong ACN Ashbrook AW. Male germ cells support long-term propagation of Zika virus. Nat Commun. 2018;9:2090. 29844387 10.1038/s41467-018-04444-w PMC5974187

[42] Brochado-Kith O Martínez I Berenguer J. HCV cure with direct-acting antivirals improves liver and immunological markers in HIV/HCV-coinfected patients. Front Immunol. 2021;12:723196. 34497613 10.3389/fimmu.2021.723196 PMC8419228

[43] Huang WC Tung S-L Chen Y-L. IFI44L is a novel tumor suppressor in human hepatocellular carcinoma affecting cancer stemness, metastasis, and drug resistance via regulating met/Src signaling pathway. BMC Cancer. 2018;18:609. 29848298 10.1186/s12885-018-4529-9 PMC5977745

[44] Kurji KH Cui JZ Lin T. Microarray analysis identifies changes in inflammatory gene expression in response to amyloid-beta stimulation of cultured human retinal pigment epithelial cells. Invest Ophthalmol Vis Sci. 2010;51:1151–63. 19797223 10.1167/iovs.09-3622 PMC3947389

[45] Vincent A Munier FL Vandenhoven CC. The characterization of retinal phenotype in a family with C1QTNF5-related late-onset retinal degeneration. Retina. 2012;32:1643–51. 22277927 10.1097/IAE.0b013e318240a574

[46] Chekuri A Zientara-Rytter K Soto-Hermida A. Late-onset retinal degeneration pathology due to mutations in CTRP5 is mediated through HTRA1. Aging Cell. 2019;18:e13011. 31385385 10.1111/acel.13011 PMC6826137

[47] Schäffler A Buechler C. CTRP family: linking immunity to metabolism. Trends Endocrinol Metab. 2012;23:194–204. 22261190 10.1016/j.tem.2011.12.003

[48] Yan Z Zhao J Gan L. CTRP3 is a novel biomarker for diabetic retinopathy and inhibits HGHL-induced VCAM-1 expression in an AMPK-dependent manner. PLoS One. 2017;12:e0178253. 28632765 10.1371/journal.pone.0178253 PMC5478095

[49] Rostamkhani H Mellati AA Tabaei BS. Association of serum zinc and vitamin A levels with severity of retinopathy in type 2 diabetic patients: a cross-sectional study. Biol Trace Elem Res. 2019;192:123–8. 30790120 10.1007/s12011-019-01664-z

[50] Speicher MA Danis RP Criswell M. Pharmacologic therapy for diabetic retinopathy. Expert Opin Emerg Drugs. 2003;8:239–50. 14610924 10.1517/14728214.8.1.239

[51] Yeung YT Aziz F Guerrero-Castilla A. Signaling pathways in inflammation and anti-inflammatory therapies. Curr Pharm Des. 2018;24:1449–84. 29589535 10.2174/1381612824666180327165604

