# Supplementary material for: IFI44L and C1QTNF5 as promising biomarkers of proliferative diabetic retinopathy
Source: Medicine (Baltimore). 2022 Nov 25;101(47):e31961. doi: 10.1097/MD.0000000000031961 (PMC9704899; doi:10.1097/MD.0000000000031961)
Supplement: Supplementary file 1 [file medi-101-e31961-s001.pdf]

Table S1 Statistic of gene modules constructed by WGCNA in GSE64036

ID	Color	Module size	Cor. with PDR
1	Black	200	0.19
2	Blue	424	0.8
3	Brown	422	0.67
4	Green	282	-0.33
5	Greenyellow	62	0.35
6	Grey	19	-0.074
7	Magenta	142	-0.054
8	Pink	170	0.77
9	Purple	76	0.18
10	Red	231	-0.1
11	Salmon	44	-0.15

12	Tan	53	-0.76
13	Turquoise	2568	-0.41
14	Yellow	307	-0.57

WGCNA: weighted gene co-expression network analysis. Cor. with PDR: Correlation coefficient with proliferative diabetic retinopathy.
