# Supplementary material for: IFI44L and C1QTNF5 as promising biomarkers of proliferative diabetic retinopathy
Source: Medicine (Baltimore). 2022 Nov 25;101(47):e31961. doi: 10.1097/MD.0000000000031961 (PMC9704899; doi:10.1097/MD.0000000000031961)
Supplement: Supplementary file 2 [file medi-101-e31961-s002.pdf]

Table S2 Gene Ontology (GO) terms enrichment analysis of the blue module

Category	Term ID	Description	Enrichment score	*p* value
Biology process	GO:0043328	protein transport to vacuole involved in ubiquitin-dependent protein catabolic process via the multivesicular body sorting pathway	32.3	0.0014
	GO:0002318	myeloid progenitor cell differentiation	25.84	0.0023
	GO:0045040	protein import into mitochondrial outer membrane	25.84	0.0023
	GO:0060161	positive regulation of dopamine receptor signaling pathway	25.84	0.0023
	GO:0032511	late endosome to vacuole transport via multivesicular body sorting pathway	24.23	0.0002
	GO:0009136	purine nucleoside diphosphate biosynthetic process	21.53	0.0034
	GO:0009180	purine ribonucleoside diphosphate biosynthetic process	21.53	0.0034

	GO:0009188	ribonucleoside diphosphate biosynthetic process	21.53	0.0034
	GO:0032020	ISG15-protein conjugation	21.53	0.0034
	GO:0045915	positive regulation of catecholamine metabolic process	21.53	0.0034
Molecular function	GO:0016508	long-chain-enoyl-CoA hydratase activity	30.72	0.0015
	GO:0030060	L-malate dehydrogenase activity	30.72	0.0015
	GO:0042296	ISG15 transferase activity	30.72	0.0015
	GO:0000822	inositol hexakisphosphate binding	20.48	0.0480
	GO:0001605	adrenomedullin receptor activity	20.48	0.0480
	GO:0003872	6-phosphofructokinase activity	20.48	0.0480
	GO:0003994	aconitate hydratase activity	20.48	0.0480
	GO:0004142	diacylglycerol cholinephosphotransferase activity	20.48	0.0480
	GO:0004307	ethanolaminephosphotransferase activity	20.48	0.0480

	GO:0004332	fructose-bisphosphate aldolase activity	20.48	0.0480
Cellular component	GO:0061574	ASAP complex	33.08	0.0013
	GO:0000243	commitment complex	26.47	0.0022
	GO:0097422	tubular endosome	26.47	0.0022
	GO:0000814	ESCRT II complex	22.05	0.0447
	GO:0005945	6-phosphofructokinase complex	22.05	0.0447
	GO:0031084	BLOC-2 complex	22.05	0.0447
	GO:0033263	CORVET complex	22.05	0.0447
	GO:0034751	aryl hydrocarbon receptor complex	22.05	0.0447
	GO:0035060	brahma complex	22.05	0.0447
	GO:0043260	laminin-11 complex	22.05	0.0447
